# Pro-oncogene FBI-1 inhibits the ferroptosis of prostate carcinoma PC-3 cells via the microRNA-324-3p/GPX4 axis

**DOI:** 10.7150/jca.96306

**Published:** 2024-06-01

**Authors:** Mingsheng Liu, Chenxiang Xu, Hua Yang, Qiyu Jiang, Guanyu Chen, Wei Wang, Tao Shao, Tibin Deng, Fei Yuan, Pingbo Xie, Hongqing Zhou

**Affiliations:** 1 Second Ward of Urology, Qujing Affiliated Hospital of Kunming Medical University, Qujing City 655000, Yunnan Province, People's Republic of China.; 2 Department of the Medical Oncology / the Hebei Key Laboratory of the Cancer Radiotherapy and Chemotherapy; the Affiliated Hospital of Hebei University; Baoding City 071000, Hebei province, People's Republic of China.; 3 Institute of Infectious Diseases, Department of Infectious Diseases, Fifth Medical Center of Chinese PLA General Hospital, 100 Middle Street of 4th West Ring Road, Beijing, 100039, China.

**Keywords:** factor that binds to the inducer of short transcripts-1, glutathione peroxidase 4, miRNA-324-3p

## Abstract

Ferroptosis has been characterized as non-apoptotic programmed cell death and is considered a novel strategy for antitumor treatment. The factor that binds to inducer of short transcripts‐1 (FBI-1) is an important proto‐oncogene playing multiple roles in human malignancies and the development of resistance to therapy. However, the roles of FBI-1 in ferroptosis of endocrine independent prostate carcinoma are still unknown. The results of this study showed that FBI-1 inhibited the ferroptosis of prostate carcinoma PC-3 cells (a typical endocrine-independent prostate carcinoma cell line) via the miR-324-3p/glutathione peroxidase 4 (miR-324-3p/GPX4) axis. Overexpression of FBI-1 enhanced the expression levels of GPX4. In contrast, knockdown of FBI-1 decreased the expression of GPX4 and induced the ferroptosis of PC-3 cells. The miR-324-3p decreased the expression of GPX4 by targeting the 3'-untranslated region of GPX4 to induce ferroptosis. Notably, FBI-1 increased the expression of GPX4 by repressing the levels of miR-324-3p. The transcription of miR-324-3p was mediated by specificity protein 1 (SP1), and FBI-1 repressed the expression of miR-324-3p by repressing the activation of SP1. In clinical specimens, the endogenous levels of FBI-1 were positively associated with Glutathione Peroxidase 4 (GPX4) and negatively related with the expression of miR-324-3p. Therefore, the results indicated that the miR-324-3p/GPX4 axis participates in the FBI-1-mediated ferroptosis of prostate carcinoma cells.

## 1. Introduction

Ferroptosis is a type of programmed cell death that differs from apoptosis, necrosis, or pyroptosis, and has attracted considerable research attention 1,2. Biochemically, ferroptosis is often characterized by the accumulation of intracellular iron ions or lipid peroxides. The occurrence and development of cancer are closely related to ferroptosis 3-5. Induction of ferroptosis is also considered a novel and promising therapeutic strategy for the treatment of malignancies 6. In cancerous cells, ferroptosis is regulated by certain signaling pathways, such as metabolism- and redox homeostasis-related pathways 5,6. Among these pathways, glutathione peroxidase 4 (GPX4) is one of the most important regulators/inhibitors of ferroptosis. This pathway inhibits the lipid peroxidation and reduces highly toxic lipid peroxides to protect cells from oxidative damage through glutathione catalysis. Inhibition of GPX4 inhibits the ability of cancerous cells to scavenge lipid peroxides and induces ferroptosis 7,8. Therefore, it is valuable to reveal the mechanism involved in the regulation of GPX4 in human cancer cells.

The pro-oncogene FBI‐1 (factor that binds to inducer of short transcripts‐1) is also termed the leukemia/lymphoma‐related factor (LRF), osteoclast‐derived zinc finger (OCZF), POK erythroid myeloid ontogenic factor (Pokemon), or zinc finger and BTB domain-containing protein 7A (ZBTB7A). It plays important roles in the occurrence and progression of multiple types of human cancer, as well as the development of drug resistance 9. FBI-1 can promote the proliferation or metastasis of cancer cells by repressing tumor suppressors and induce resistance to antitumor agents. Evidence has revealed that FBI-1 is a promising and valuable target for the treatment of malignancies, including lung cancer, hepatocellular carcinoma, breast cancer, and colorectal cancer 10-12. Therefore, it is necessary to investigate the role of FBI-1 in other types of cancer, particularly prostate cancer.

Currently, the overall prognosis for endocrine-independent prostate carcinoma/castration-resistant prostate cancer (CRPC) remains poor due to its insensitivity to androgen deprivation therapy and highly aggressive nature 13,14. It is important to elucidate the mechanisms regulating CRPC, specifically focusing on the molecular alterations that participate in the activation of androgen receptor-independent pathways; such knowledge may lead to the development of novel therapeutic strategies 14,15. This the first study demonstrating that FBI-1 regulates cellular iron death via the miRNA-324-3p/GPX4 axis in PC-3 cells. These results expand our understanding of FBI-1 and provide a solid foundation for a new strategy based on iron death for the treatment of CRPC.

## 2. Results

### 2.1. Knockdown of FBI-1 induced the ferroptosis of PC-3 cells

**Figure 1:**
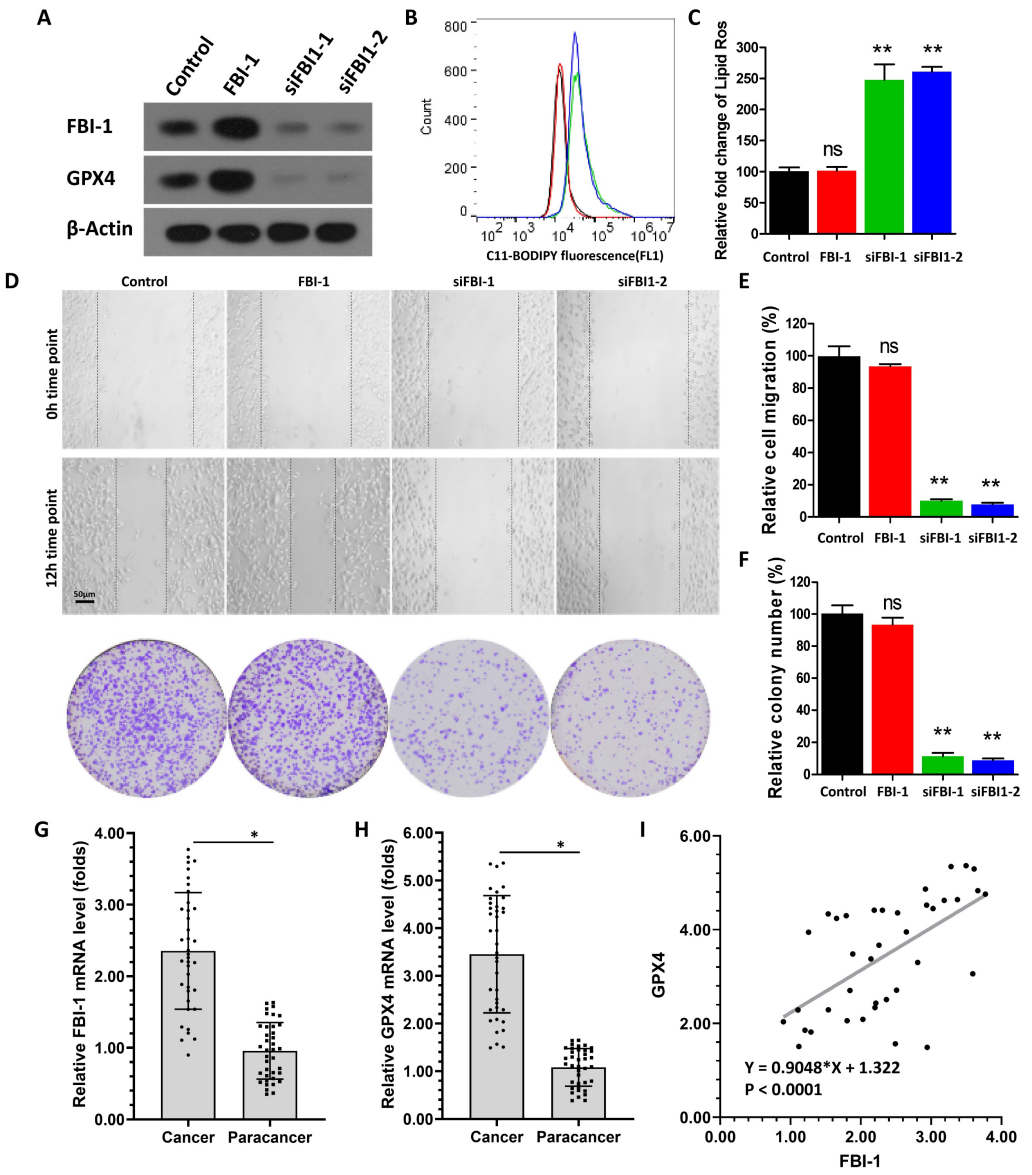
Knockdown of FBI-1 induced the ferroptosis of PC-3 cells. (A) The PC-3 cells were transfected with the vectors (Control, FBI-1, siFBI1-1 or siFBI1-2) and harvested for western blotting. Western blotting images show the expression levels of FBI-1 and GPX4. β-actin was selected as the loading control. (B and C) PC-3 cells were transfected with the vectors and harvested for flow cytometry. The levels of lipid peroxidation/cytosolic ROS in PC-3 cells are shown as images obtained from flow cytometry (B) or quantitative results (C). (D-F) PC-3 cells were analyzed using wound-healing and colony formation assays. Results are shown as images (D) and quantitative results (E and F). The expression levels (endogenous mRNA) of FBI-1 (G) and GPX4 (H) in PC clinical specimens are shown as scatter-bar images (G and H), and the correlation between FBI-1 and GPX4 in PC tissues (I) was determined. Abbreviations: FBI-1, factor that binds to inducer of short transcripts‐1; GPX4, glutathione peroxidase 4; PC, prostate carcinoma; ROS, reactive oxygen species.

Firstly, we investigated the effect of FBI-1 on the ferroptosis of PC-3 cells. As shown in Figure 1A, overexpression of FBI-1 enhanced the expression of GPX4 in PC-3 cells. In contrast, knockdown of FBI-1 by siRNAs (FBI-1 siRNA 1 [siFBI-1] or FBI-1 siRNA 2 [siFBI-2]) decreased the expression of GPX4 in PC-3 cells. Moreover, overexpression of FBI-1 did not modulate the ferroptosis (lipid peroxidation/cytosolic ROS) of PC-3 cells (Figures 1B and C); however, the levels of lipid peroxidation/cytosolic ROS in PC-3 cells were not affected by FBI-1 overexpression (Figure 1B and C). Knockdown of FBI-1 induced the ferroptosis of PC-3 cells (Figure 1B and C). The levels of lipid peroxidation/cytosolic ROS in PC-3 cells were significantly increased after FBI-1 knockdown by siFBI-1 or siFBI-2 (Figures 1B and C). Moreover, overexpression of FBI-1 enhanced the proliferation (colony formation assay) and migration (wound-healing assay) of PC-3 cells (Figures 1D-F). In contrast, knockdown of FBI-1 repressed the proliferation and migration of PC-3 cells (Figures 1D-F). These results indicated that FBI-1 modulated the ferroptosis of PC-3 cells.

To further examine the effect of FBI-1 on GPX4, their expression levels were examined in clinical specimens obtained from patients with CRPC. As shown in Figures 1H and I, the expression of FBI-1 was significantly higher in CRPC tissues versus para-cancerous tissues (paired non-tumor tissues) (Figure 1G). The expression of GPX4 was also significantly higher in CRPC tissues versus para-cancerous tissues (Figure 1H). In addition, in tissue specimens, the expression levels of FBI-1 were positively correlated with those of GPX4 (Figure 1I). These results further supported the effect of FBI-1 on GPX4.

### 2.2. FBI-1 enhanced the expression of FBI-1 by suppressing that of miRNA-324-3p in PC-3 cells

**Figure 2:**
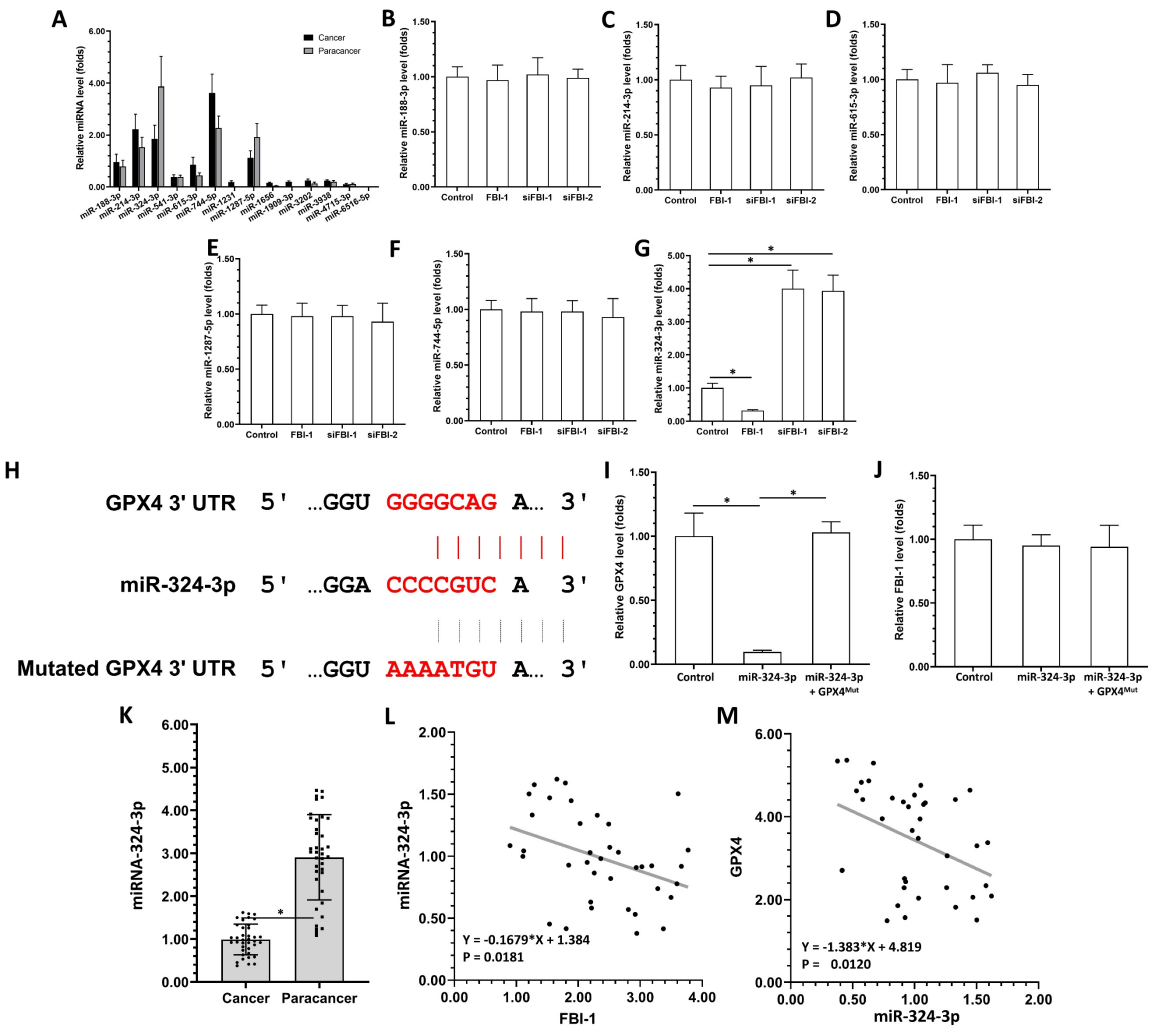
The miRNA-324-3p repressed the expression of GPX4 in PC-3 cells by targeting its mRNA 3'UTR. (A) The expression levels of miRNAs (miRNA-188-3p, miRNA-214-3p, miR-324-3p, miR-541-3p, miRNA-615-3p, miRNA-744-5p, miRNA-1231, miRNA-1287-5p, miRNA-1656, miRNA-1909-3p, miRNA-3202, miRNA-3938, miRNA-4715-3p, or miRNA-6516-5p) potentially targeting GPX4 were examined in clinical specimens (PC tissues or paired para-cancerous non-tumor tissues) through qPCR. (B-G) PC-3 cells were transfected with control, FBI-1, or siRNA of FBI-1 (siFBI1-1 or siFBI1-2). The levels of miRNAs in PC-3 cells were examined by qPCR to reveal the effect of FBI-1 on their expression. (H) The targeting site of miRNA-324-3p in the 3'UTR of GPX4 mRNA and the GPX4 with the mutated targeting site of miRNA-324-3p in the 3'UTR (termed GPX4^Mut^). (I and H) PC-3 cells were transfected with control, miRNA-324-3p, or miRNA-324-3p + GPX4^Mut^. The expression levels of GPX4 and FBI-1 were examined by qPCR to determine the effect of miRNA-324-3p on GPX4 and FBI-1 in PC-3 cells. Abbreviations: FBI-1, factor that binds to inducer of short transcripts‐1; GPX4, glutathione peroxidase 4; PC, prostate carcinoma; qPCR, quantitative polymerase chain reaction; siFBI1-1, FBI-1 siRNA 1; siFBI1-2, FBI-1 siRNA 2; UTR, untranslated region.

The above results demonstrated that FBI-1 can modulate the ferroptosis of PC-3 cells by enhancing the expression of GPX4. The presence of miRNAs potentially targeting GPX4 was examined in the clinical specimens to identify the mechanism regulating the expression of GPX4. As shown in Figure 2A, the selected miRNAs (i.e., miRNA-1231, miRNA-1656, miRNA-1909-3p, miRNA-3202, miRNA-3938, miRNA-4715-3p, and miRNA-6516-5p) were not expressed or expressed at extremely low levels in clinical tissues (Figure 2A), including tumor and para-cancerous tissues (Figure 2A). The expression of several other miRNAs, miRNA-188-3p and miRNA-541-3p, did not differ significantly between tumor tissues and para-cancerous tissues. However, the expression of miRNA-214-3p and miRNA-615-3p was markedly higher in tumor tissues versus para-cancerous tissues (Figure 2A). The miRNA-324-3p was highly expressed in para-cancerous tissues compared with tumor tissues (Figure 2A). Subsequently, the effects of FBI-1 on the miRNAs were further examined. As shown in Figures 2B-G, overexpression of FBI-1 decreased the levels of miRNA-324-3p in PC-3 cells, whereas and knockdown of FBI-1 increased the levels of miRNA-324-3p in PC-3 cells. Overexpression or knockdown of FBI-1 did not alter the expression of miR-188-3p, miR-214-3p, miR-615-3p, miR-1287-5p or miR-744-5p in PC-3 cells (Figures 2B-G). These results showed that FBI-1 could enhance the expression of GPX4 by suppressing that of miRNA-324-3p in PC-3 cells.

### 2.3. The miRNA-324-3p repressed the expression of GPX4 in PC-3 cells

The above results showed that miRNA-324-3p could target GPX4 and be suppressed by FBI-1 in PC-3 cells. The effect of miRNA-324-3p on the expression of GPX4 was further evaluated in PC-3 cells. The sequences of miRNA-324-3p and the target site of miRNA-324-3p in the 3'-untranslated region (3'UTR) of GPX4 are shown as Figure 2H. Overexpression of miRNA-324-3p decreased the expression of wild-type GPX4 in PC-3 cells (Figure 2I). However, it did not affect the levels of GPX4 with a mutated miRNA-targeting site in the 3'UTR (Figure 2I). The expression of GPX4 was rescued after co-transfection with miRNA and GPX4^mut^ (the expression vector of GPX4 with a mutated miRNA-targeting site) (Figure 2I). The specificity of miRNA-324-3p was further confirmed by the results demonstrating that overexpression of miRNA-324-3p and transfection with GPX4^mut^ did not influence the expression of FBI-1 (Figure 2J).

Next, the correlation between the expression levels of GPX4 and miRNA-324-3p in clinical specimens was examined. As shown in Figure 2K, the expression of miRNA-324-3p was markedly lower in CRPC clinical specimens compared with non-tumor tissues. Moreover, the expression of miRNA-324-3p was negatively related with GPX4 in CRPC clinical specimens (Figure 2K). The expression of FBI-1 was positively related with GPX4 and negatively related with miRNA-324-3p (Figures 2L and M). Therefore, the FBI-1/miRNA-324-3p axis may be involved in the aberrant expression of GPX4 in prostate cancer.

### 2.4. Specificity of the FBI-1/miRNA-324-3p/GPX4 axis in the regulation of ferroptosis of PC-3 cells

**Figure 3:**
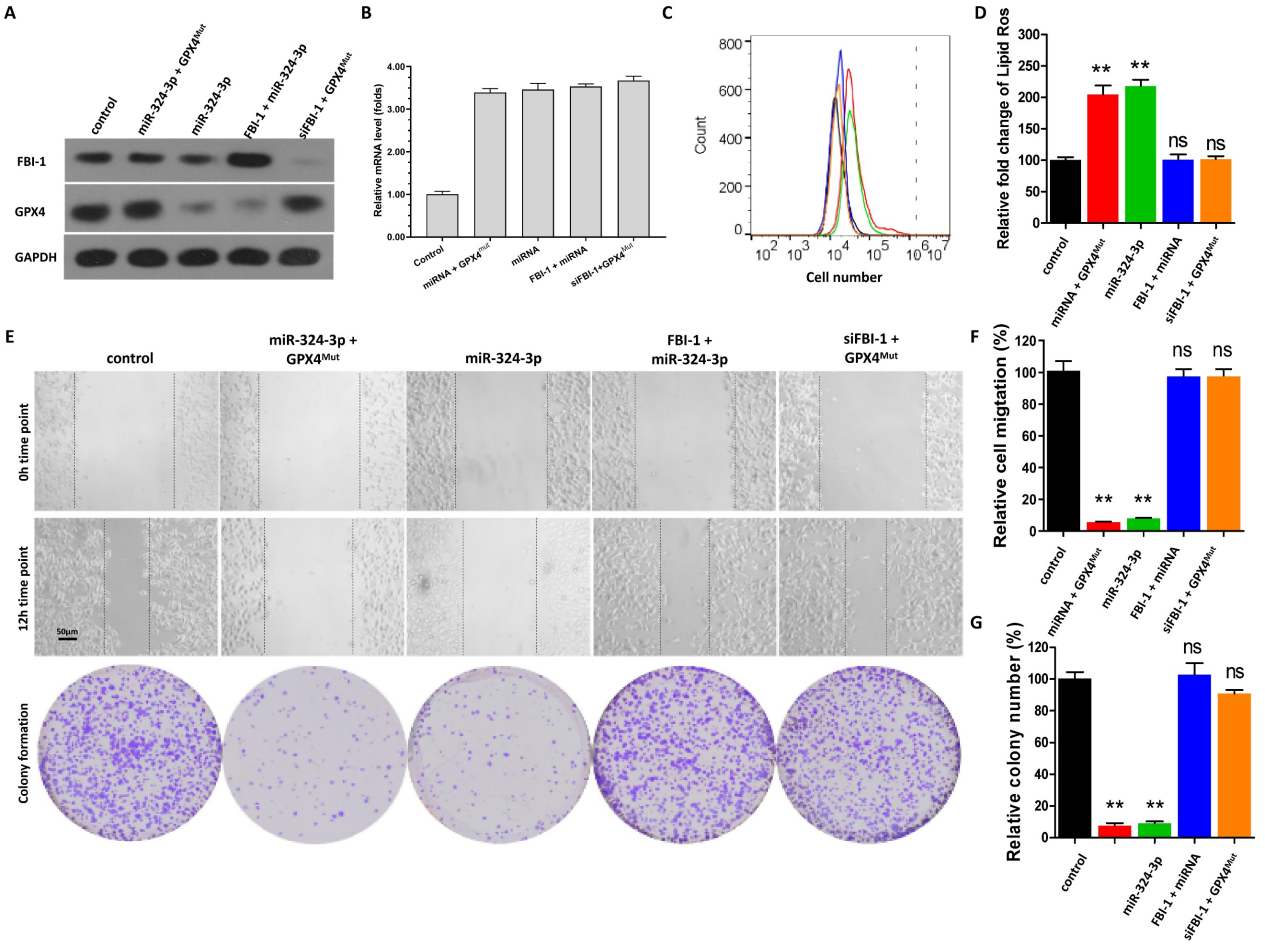
Specificity of the FBI-1/miRNA-324-3p/GPX4 axis in ferroptosis of PC-3 cells. PC-3 cells were transfected with vectors (control, miRNA-324-3p + GPX4^Mut^, miRNA-324-3p, FBI-1 + miRNA-324-3p, or siFBI-1 + GPX4^Mut^). (A) PC-3 cells were harvested for western blotting analysis to examine the expression of FBI-1 and GPX4. (B) PC-3 cells were harvested for qPCR to examine the expression of miRNA-324-3p. (C and D) The cells were harvested for flow cytometry to examine the levels of lipid peroxidation/cytosolic ROS. (E-G) PC-3 cells were harvested for wound-healing and colony formation assays to examine the effect of the FBI-1/miRNA-324-3p/GPX4 axis on the proliferation of PC-3 cells. *P<0.05 compared with the control group. Abbreviations: FBI-1, factor that binds to inducer of short transcripts‐1; GPX4, glutathione peroxidase 4; qPCR, quantitative polymerase chain reaction; ROS, reactive oxygen species. \

The effect of the FBI-1/miRNA-324-3p/GPX4 axis on the ferroptosis of PC-3 cells was examined to confirm the specificity of FBI-1 on GPX4. In PC-3 cells, overexpression of miRNA-324-3p alone significantly downregulated the protein expression of GPX4; however, it did not affect the levels of FBI-1 (Figures 3A and B). Transfection with GPX4^Mut^ during the overexpression of miRNA-324-3p rescued the expression of GPX4 in PC-3 cells (Figures 3A and B). Overexpression of miRNA-324-3p along with overexpression of FBI-1 in PC-3 cells (Figures 3A and B) blocked the induction of GPX4 expression by FBI-1 in PC-3 cells (Figures 3A and B). Finally, transfection with GPX4^Mut^ and knockdown of FBI-1 inhibited the downregulation of GPX4 expression by FBI-1 (Figures 3A and B).

Moreover, overexpression of miRNA-324-3p alone induced the ferroptosis of PC-3 cells (Figure 3C and D). Transfection with GPX4^Mut^ during the overexpression of miRNA-324-3p almost blocked the induction of ferroptosis in PC-3 cells by miRNA-324-3p (Figures 3C and D). Overexpression of miRNA-324-3p and FBI-1 in PC-3 cells did not affect the induction of ferroptosis in PC-3 cells (Figures 3C and D). Finally, transfection of GPX4^Mut^ and knockdown of FBI-1 (siFBI-1) were performed (Figure 3C and D); knockdown of FBI-1 expression could not induce the ferroptosis of PC-3 cells at this time (Figures 3C and D). Similar results were obtained from the scratch assay as well as the cell formation colony assay (Figure 3E-G). These results further confirm the specificity of the FBI-1/miRNA/GPX4 axis for regulating the ferroptosis of PC-3 cells.

### 2.5. FBI-1 suppressed the expression of miRNA-324-3p by suppressing the activation of transcription factor SP1 in PC-3 cells

**Figure 4:**
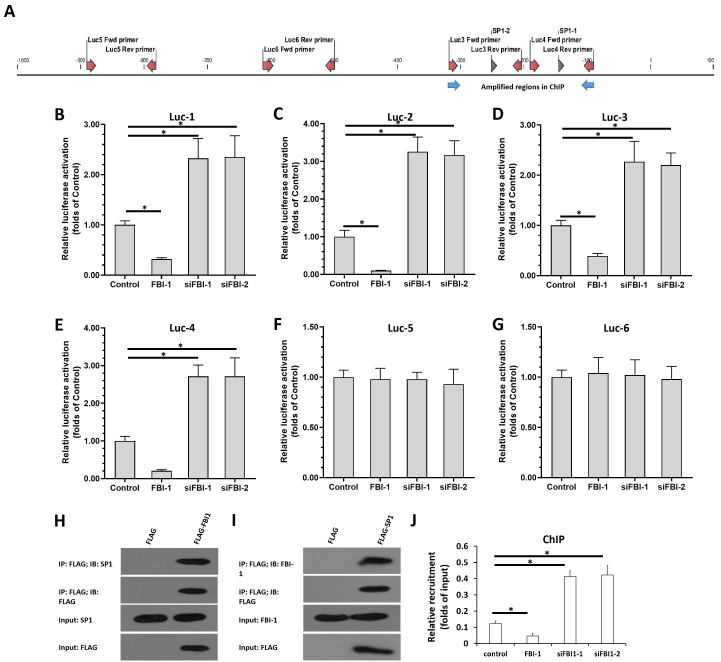
SP1 mediated the transcription of miR-324-3p, while FBI-1 repressed the transcriptional activation of SP1, as shown by luciferase reporter assays. (A) Two SP1-binding sites located in the selected promoter region of miRNA-324-3p. The two SP1-binding site pentamer sequences, the 100-bp upstream and downstream sequences of the two SP1-binding sites, and two 100-bp sequences of the miRNA-324-3p promoter region that do not contain the SP1-binding site were prepared as luciferase reporters. These reporters were termed Luc-1, Luc- 2, Luc-3, Luc-4, Luc-5, and Luc-6. (B-G) PC-3 cells were transfected with the vectors (control, FBI-1, or siFBI-1) and co-transfected with luciferase reporters (Luc-1-Luc-6). Finally, the cells were harvested for analysis via luciferase reporter assays. (H and I) The PC-3 cells were transfected with FLAG, FLAG-FBI1 (H), or FLAG-SP1 (I), and the cells were harvested for IP assays. (J) The PC-3 cells were transfected with FBI-1 or siFBI1 (siFBI1-1 or siFBI1-2) and harvested for the ChIP assays to detect the recruitment of SP1 to miRNA-324-3p's promoter. *P<0.05. Abbreviations: FBI-1, factor that binds to inducer of short transcripts‐1; Luc, luciferase; SP1, specificity protein 1; IP: Immunoprecipitation; ChIP, Chromatin immunoprecipitation.

Next, we performed multiple assays to reveal the mechanism through which FBI-1 represses the expression of miRNA-324-3p in PC-3 cells. Bioinformatics analysis was performed to analyze sequences from a region of the miR-324-3p promoter (http://tfbind.hgc.jp). As shown in Figure 4A, the miRNA promoter contained SP1-binding sites, and the results revealed that FBI-1 could repress the expression of targeting genes by interacting with SP1. As shown in Figures 4B-G, overexpression of FBI-1 suppressed the activation of luciferase reporters: Luc-1 (the luciferase reporter of the first SP1-binding site in the miRNA-324-3p promoter), Luc-2 (the luciferase reporter of the second SP1-binding site in the miRNA-324-3p promoter), Luc-3 (the luciferase reporter containing approximately 100 bp of sequence upstream and downstream of the first SP1-binding site in the miRNA-324-3p promoter), or Luc-4 (the luciferase reporter containing approximately 100 bp of sequence upstream and downstream of the second SP1-binding site in the miRNA-324-3p promoter). Knockdown of FBI-1 enhanced the activation of these luciferase reporters (Figures 4B-G). As important controls, Luc-5 and Luc-6 did not contain SP1-binding sites. As expected, overexpression or knockdown of FBI-1 expression did not affect the activity of Luc-5 and Luc-6 (Figures 4B-G). Moreover, the interaction between FBI-1 with SP1 in PC-3 cells were confirmed by the IP and re-IP assays (Figure 4H and Figure 4I). The effect of FBI-1 on SP1 was also examined by the ChIP assays (Figure 4J). As shown in Figure 4J, SP1 could recruit to the promoter region of miRNA-324-3p containing the SP1 binding sites. Overexpression of FBI-1 inhibited the recruitment of SP1 to miRNA-324-3p's promoter region; whereas knockdown of FBI-1 enhanced the recruitment of SP1 to miRNA-324-3p's promoter region in PC-3 cells (Figure 4J).

**Figure 5:**
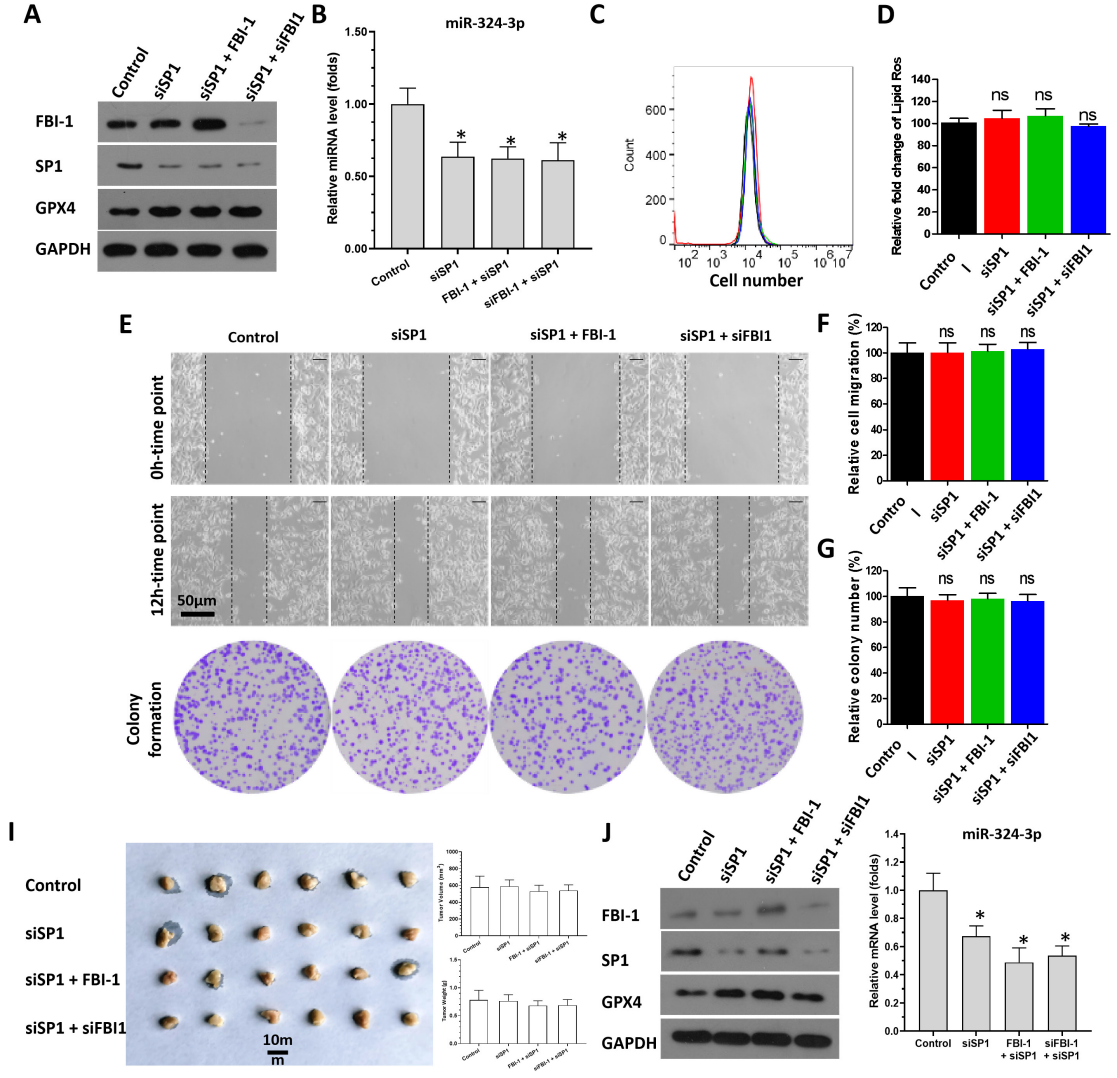
FBI-1 repressed the expression levels of miRNA-324-3p by SP1. PC-3 cells were transfected with vectors (control, siSP1, FBI-1, or siFBI-1). (A) Cells were harvested for western blotting to determine the protein levels of FBI-1, SP1, and GPX4. (B) Cells were harvested for qPCR to measure the levels of miRNA-324-3p. (C and D) Cells were harvested for flow cytometry to examine the levels of lipid peroxidation/cytosolic ROS. (E-G) PC-3 cells were harvested for wound-healing and colony formation assays to investigate the effect of the FBI-1/SP1/miRNA-324-3p axis on the proliferation of PC-3 cells. The cells of Figure 5A was injected into nude mice to form subcutaneous tumor tissues (I). The results were shown as images of tumors, tumor volumes or tumor tissues (I). The expression of GPX4, FBI-1, SP1 or miRNA-324-3p in the tumor tissues (mixed samples of tumor tissue from each group) was examined by western blot or qPCR (J). Abbreviations: FBI-1, factor that binds to inducer of short transcripts‐1; GPX4, glutathione peroxidase 4; Luc, luciferase; qPCR, quantitative polymerase chain reaction; ROS, reactive oxygen species; siFBI1-1, FBI-1 siRNA 1; SP1, specificity protein 1.

SP1 was knocked down in PC-3 cells to further confirm the specificity of FBI-1/SP1 on the miRNA-324-3p /GPX4 axis (Figure 5). As shown in Figure 5A, use of siRNA significantly reduced SP1 expression in PC-3 cells. In contrast, knockdown of SP1 expression did not affect the levels of FBI-1 (Figure 5A). As expected, knockdown of SP1 expression in PC-3 cells upregulated the expression of miRNA-324-3p (Figure 5B). Thereafter, neither overexpression of FBI-1 in PC-3 cells nor knockdown of FBI-1 expression levels could not affect the expression of GPX4, miRNA-324-3p or the ferroptosis of PC-3 cells on the basis of knockdown of SP1 (Figures 5A and B). Moreover, ferroptosis was significantly induced in PC-3 cells after knockdown of SP1 expression using siRNA (Figures 5C and D). At this point, neither overexpression nor knockdown of FBI-1 expression could affect ferroptosis in PC-3 cells (flow cytometry results) (Figures 5C and D). The wound-healing and colony formation assays yielded similar findings to those obtained through flow cytometry (Figures 5E-G). We also examined the effect of SP1 knockdown and SP1 knockdown followed by overexpression or knockdown of FBI-1 on miRNA expression as a control. Knockdown of SP1 expression downregulated miRNA-324-3p expression in PC-3 cells. However, knockdown of SP1 followed by FBI-1 overexpression or knockdown was no longer able to affect miRNA-324-3p expression (Figure 5B). These results were obtained from the *in vitro* assays and the *in vivo* assays were also performed (Figure 5I and J). In subcutaneous tumor tissues (Figure 5I), knockdown of SP1 decreased the expression of miRNA-324-3p and enhanced the expression of GPX4 (Figure 5J). When SP1 had been knocked down, neither overexpression of FBI-1 nor knockdown of FBI-1 had any effect on miRNA-324-3p or GPX4 in the tissue at this point (Figure 5J). These results indicate that FBI-1 suppresses the expression of miRNA-324-3p by suppressing the activation of transcription factor SP1 in PC-3 cells.

### 2.6. *In vivo* effect of FBI-1 on the miRNA/GPX4 axis in PC-3 cells

**Figure 6:**
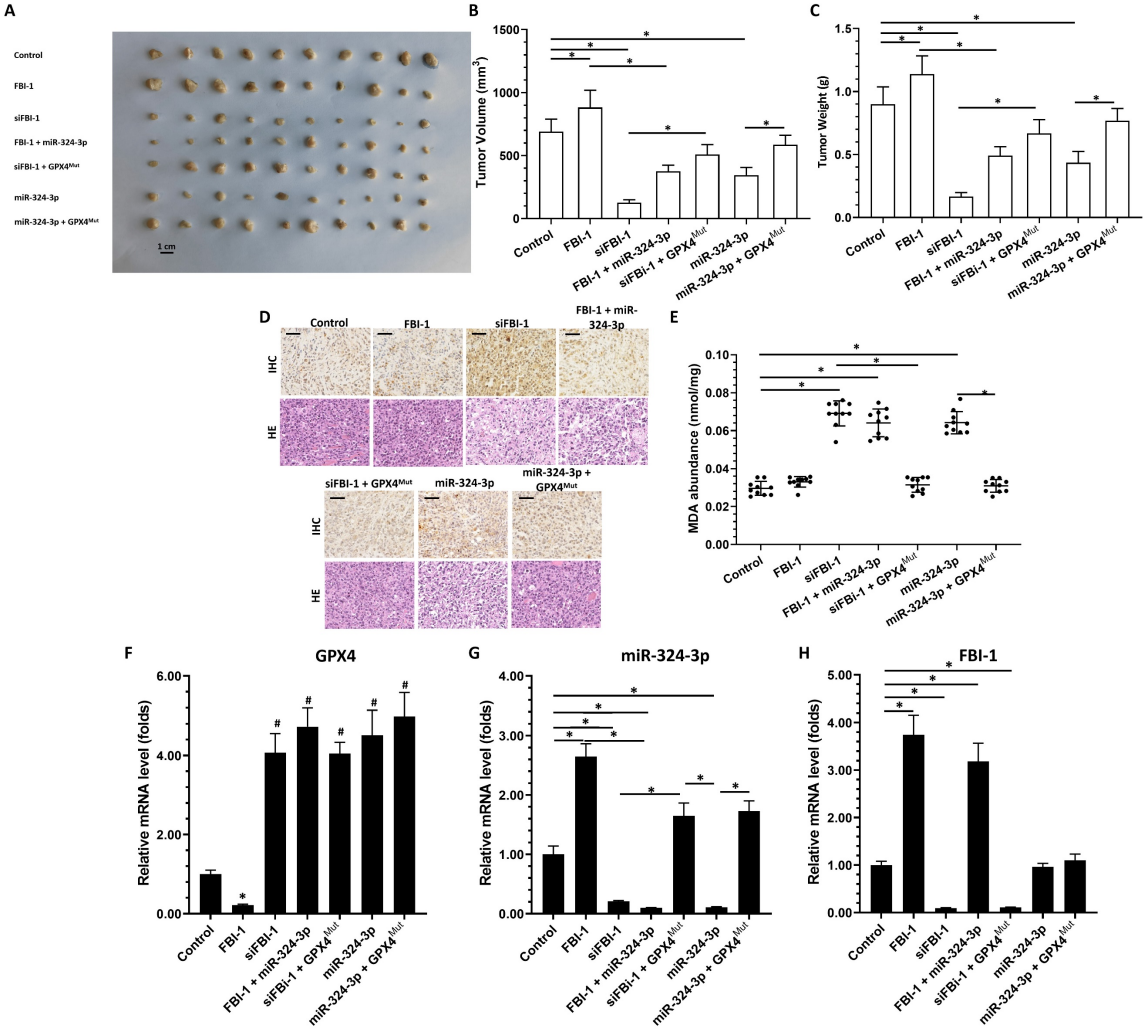
Effect of FBI-1 on the miRNA-324-3p/GPX4 axis in subcutaneous tumor tissues. PC-3 cells were transfected with vectors and subcutaneously injected into nude mice. (A) Images of subcutaneous tumor tissues formed by the PC-3 cells. (B) Tumor volumes. (C) Tumor weights. (D and E) Representative images of 4-HNE IHC staining and H&E of subcutaneous tumors harvested from each group (scale bars: 50 μm) (D), and the levels of MDA (E) in each group of tumor tissues. (F-H) Tumor tissues were harvested for qPCR to examine the expression of GPX4 (F), miRNA-324-3p (G), and FBI-1 (H). *P<0.05. Abbreviations: 4-HNE, 4-hydroxynonenal; FBI-1, factor that binds to inducer of short transcripts‐1; GPX4, glutathione peroxidase 4; H&E, hematoxylin and eosin; IHC, immunohistochemical; MDA, malondialdehyde.

As shown in Figure 6, PC-3 cells could form subcutaneous tumor tissues in nude mice. Overexpression of FBI-1 promoted the tumorigenic effect of PC-3 cells in the subcutis of nude mice, upregulated the expression of GPX4 and downregulated that of miRNA-324-3p in tumor tissues (Figure 6A-C). Knockdown of FBI-1 expression inhibited the tumorigenic effect of PC-3 cells under the skin of nude mice, and downregulated the expression of GPX4 and miRNA-342-3p in tumor tissues (Figure 6A-C). In PC-3 cells overexpressing FBI-1 along with miRNA-342-3p, FBI-1 could no longer affect the expression of GPX4 (Figure 6A-C). Following knockdown of FBI-1 and overexpression of GPX4^Mut^, knockdown of FBI-1 remained able to upregulate miRNA-324-3p expression (Figure 6A-C). However, miRNA-324-3p could no longer inhibit the expression of GPX4^Mut^ (Figure 6A-C). The results were shown as images of subcutaneous tumor tissues (Figure 6A), and the quantitative results of images (Figure 6B and C). Furthermore, overexpression of miRNA-324-3p inhibited the subcutaneous tumorigenic effect of PC-3 cells in nude mice and suppressed GPX4 expression in tumor tissues. Simultaneous overexpression of GPX4^Mut^ blocked the inhibitory effect of miRNA-342-3p on GPX4 in PC-3 cells (Figure 6A-C). The effect of FBI-1 and others on ferroptosis of PC-3 cells in tumor tissues was ultimately demonstrated by the results of the immunohistochemical analysis (Figures 6D and E show the lipid peroxide 4-HNE-stained immunohistochemical sections of tumors). These results are shown as the expression of each factor in the FBI-1/miRNA/GPX4 axis in different groups of tumor tissues (Figures 6F-H).

### 2.7. FBI-1/miR-324-3p/GPX4 in LNCaP cells

The above results mainly focused on the PC-3 cells. To further confirm the effect of FBI-1, LNCaP cells, an AR positive prostate cancer cell line, was also used. As shown in supplemental Figure 1, overexpression of FBI-1 enhanced the expression of GPX4 in LNCaP cells. Knockdown of FBI-1 by siRNAs (FBI-1 siRNA 1 [siFBI-1]) decreased the expression of GPX4. Moreover, overexpression of FBI-1 did not modulate the expression of AR in LNCaP cells (Supplemental Figure 2). These results further confirm the effect of FBI-1 in prostate cancer.

### 2.8. Effect of FBI-1 on some factors in PC-3 cells

The effect of FBI-1 on ACSL4, LPCAT3, TfR1, SLC7A11 or FTH1 in PC-3 cells were examined. As shown in supplemental Figure 2, overexpression of FBI-1 decreased the expression of ACSL4 but not LPCAT3, TfR1, SLC7A11 or FTH1. Knockdown of FBI-1 by siRNAs (FBI-1 siRNA 1 [siFBI-1]) increased the expression of ACSL4, but not LPCAT3, TfR1, SLC7A11 or FTH1 (Supplemental Figure 2). These results further confirm the effect of FBI-1 in prostate cancer.

## 3. Discussion

Prostate cancer is the most common endocrine-dependent / related tumor in males, and one of the most frequent malignancies in males 16,17. Early studies have concluded that endocrine-related therapy is important for patients with prostate cancer and that the overall prognosis for such patients should be favorable 16,17. However, the clinical management of prostate cancer continues to face numerous challenges. Firstly, surgery is a radical treatment strategy for prostate cancer that can have a significant impact on the overall survival of patients. However, some patients are diagnosed with advanced-stage disease and are thus ineligible for surgical treatment 18. Secondly, recent studies have concluded that prostate cancer is heterogeneous and that there are significant differences in the dependence of prostate cancer cells on hormones. Hormone stripping therapy is similar to clonal selection; this is inconsistent with the earlier belief that prostate cancer undergoes cellular changes during androgen antagonism therapy and eventually changes from an endocrine-dependent to a non-dependent status. This ultimately allows endocrine non-dependent or hormone stripping therapy-insensitive cells to gradually expand, thereby leading to disease progression. Patients with endocrine-independent prostate cancer (i.e., disease that has progressed after androgen antagonist therapy) are associated with a poor prognosis due to the extreme insensitivity of cells to antitumor agents (e.g., chemotherapies) compared with other kinds of malignant tumors, such as lung or liver cancer 19. This highlights the importance of research on endocrine-independent prostate cancer. In this setting, second-line therapies, genetic testing after tissue biopsy, and treatment with tyrosine kinase inhibitors (TKIs), including angiogenesis inhibitors alone or in combination with endostatin, linomide, and lapachone, have been used 20,21. However, the overall efficacy of these therapeutic strategies has not been satisfactory. Hence, identification and elucidation of the molecular mechanisms regulating endocrine non-dependent prostate cancer is urgently necessary.

**Figure 7:**
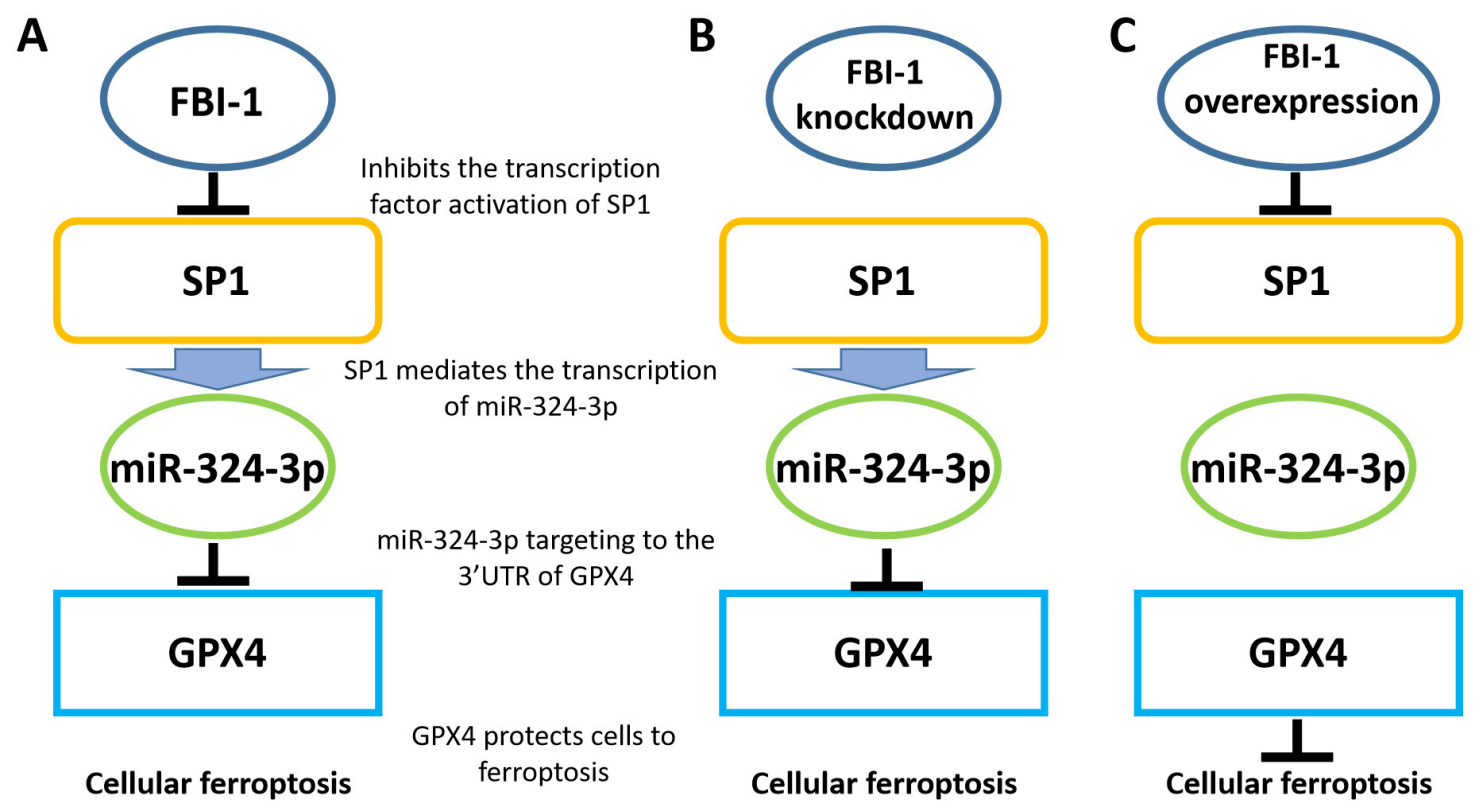
Proposed model based on the results of the present study. (A) In PC-3 cells, FBI-1 downregulated the expression of miRNA-324-3p by suppressing the transcription factor activity of SP1. FBI-1 downregulated the expression of miRNA-324-3p and upregulated that of GPX4 to regulate iron death in PC-3 cells. (B) Following the knockdown of FB-1 expression in PC-3 cells, SP1 upregulated the expression of miRNA-324-3p. This effect ultimately induced iron death in PC-3 cells by downregulating the expression of GPX4. (C) In PC-3 cells, overexpression of FBI-1 further downregulated the expression of miRNA-324-3p and ultimately GPX4 by downregulating the transcription factor activity of SP1. Abbreviations: FBI-1, factor that binds to inducer of short transcripts‐1; GPX4, glutathione peroxidase 4; SP1, specificity protein 1.

This is the first study to demonstrate that FBI-1 modulates the ferroptosis of PC-3 cells via the miRNA/GPX4 axis (Figure 7). The main feature of ferroptosis is the lethal accumulation of lipid peroxides induced by the oxidation of phospholipids containing polyunsaturated fatty acids 22,23. This renders GPX4 an excellent target for the treatment of malignant tumors. GPX4 inhibits iron death in malignant tumor cells, in particular the damage caused by abnormal lipid metabolism and peroxide metabolism 24. In this study, FBI-1 protected PC-3 cells from ferroptosis-related injury by upregulating GPX4 expression. Notably, knockdown of FBI-1 expression downregulated GPX4 expression and ultimately induced ferroptosis in PC-3 cells. FBI-1 is a positive regulator of proliferation, survival, and malignancy of various malignant cells. Moreover, it induces resistance of malignant cells to treatment with antineoplastic drugs 25-27. The results of this study suggest that FBI-1 modulates the ferroptosis of endocrine-independent prostate cancer PC-3 cells and revealed the molecular mechanism underlying these effects. Of note, the prostate cancer cell line used in this study (PC-3) is commonly used as a research model for endocrine-androgen receptor (AR)-unrelated PC. However, similar to triple-negative breast cancer 28,29, prostate cancer is heterogeneous. Therefore, the use of a single cell line may not be sufficiently representative. Future studies should investigate multiple types of cells, including plasmacytoid dendritic cells of CRPC.

FBI-1 could inhibit the ferroptosis of PC-3 cells by inhibiting the expression of miR-324-3p; the latter was identified as a type of miRNA targeting GPX4 in CRPC. We used the online bioinformatics tool miRDB database and conducted a search on PubMed (NCBI, USA), supplemented by our manual interpretation. Ultimately, we detected the expression levels of multiple miRNAs in CRPC tissues. Among the selected miRNAs, miRNA1656, miRNA-1231, miRNA-541-3p, miRNA-1909-3p, miRNA-3202, miRNA-3938, miRNA-4715-3p, and miRNA-6515-5p showed very low expression levels in CRPC tissue specimens. Among the few miRNAs with high expression, the levels of miRNA-188-3p, miRNA-214-3p, miRNA-615-3p, and miRNA-744-5p in tumor tissues exceeded those recorded in adjacent non-tumor tissues. Only two miRNAs (i.e., miRNA-324-3p and miRNA-1287) exhibited lower expression in tumor tissues versus paracancerous tissues. We also examined the effect of FBI-1 on the expression of these miRNAs The data showed that overexpression or knockdown of FBI-1 affected the expression of miRNA-324-3p, but not miRNA-744-5p (although its expression was higher than that of miRNA-324-3p in tumor tissues) or miRNA-1287 (its expression was lower in tumor tissues versus paracancerous tissues). Moreover, in PC-3 cells, FBI-1 inhibited the expression of miRNA-324-3p by suppressing the transcription factor activity of SP1 and ultimately miRNA-324-3p. The effect of FBI-1 on SP1/miRNA-324-3p was verified by luciferase reported assays and SP1 knockdown. FBI-1 inhibited the activity of luciferase reporters, which contain the SP1-binding site sequence located in the miRNA-324-3p promoter region. Following the knockdown of SP1, FBI-1 could no longer affect the expression of miRNA-324-3p. FBI-1 is an important repressor of the transcription factor SP1. Therefore, inhibition of the transcriptional activity of SP1 may be the mechanism through which FBI-1 exerts its effects 30-34. As important small non-coding RNA molecules, packaging miRNAs sequences in lentiviruses is an effective antitumor strategy 35,36. Furthermore, the lack of expression of some miRNAs that play a tumor suppressor role in malignant cells is also an important cause of malignancy 37,38. The results of this study showed that miRNA-324-3p was highly expressed in para-tumor tissues, demonstrating its clinical significance and specificity in prostate cancer. Also, its expression was significantly lower in CRPC tissues versus paraneoplastic tissues. The molecular mechanisms underlying the expression trends of several other miRNAs in clinical specimens should be further investigated. The present results showed that, in PC-3 cells, FBI-1 regulates miRNA-324-3p. The regulatory mechanisms of other miRNAs in this setting warrant further investigation. Finally, investigation of promoter region methylation should also be carried out in the future 39,40.

For the limitations of our study, the present study was a direct examination of the effect of FBI-1 on iron death in PC-3 cells, focusing mainly on the core pathway GPX4, which regulates iron death, and was followed up only purposively by the expression of some other factors. At the same time, we have mainly carried out studies on endocrine-independent prostate cancer, and for this reason, in the future, we will not only carry out studies in other types of prostate cancer, but also use multi-omics technology to deeply and systematically study the function and regulatory mechanism of FBI-1.

In conclusion, the findings of this study indicated that the miR-324-3p/GPX4 axis participates in the FBI-1-mediated ferroptosis of prostate carcinoma cells.

## 4. Materials and Methods

### 4.1. Clinical specimens, vectors, and cell lines

The protocol of this study was approved by the Medical Ethics Committee of the First People's Hospital (Qujing, China). The investigation focused on the analysis of clinical specimens from patients diagnosed with endocrine-independent prostate carcinoma (CRPC), who were considered cases of prostate cancer with disease progression despite continued androgen deprivation therapy. Specimen collection was performed from January 2016 to March 2020. The diagnosis, treatment, and inclusion/exclusion criteria of patients with CRPC in this study were based on the Chinese Medical Association Expert Consensus on the diagnosis and management of destructive resistant prostate cancer.

In this study, the inclusion criteria were: (1) patients receiving androgen antagonist therapy, whose serum testosterone levels were depleted (<1.7 nmol/L); (2) biochemical progression of disease, as demonstrated by three consecutive measurements of elevated prostate-specific antigen levels at intervals of ≥1 week, two consecutive increases by ≥50% versus the lowest value, and prostate-specific antigen levels >2 μg/L; or (3) imaging progression of disease, based on the presence of at least two new lesions on a bone scan or enlarged soft tissue lesions that met the criteria for solid tumor response evaluation. The patients underwent tumor resection through local anesthesia and percutaneous puncture. The tumor tissue obtained by puncture was separated from the paracancerous tissue by microdissection. The clinical specimens were used for extraction of total RNA or embedded in wax blocks. The total number of tissue specimens involved in this study was 32 pairs (i.e., tumor tissue and non-tumor tissue adjacent to the cancer from the same patient). The sample size was estimated as previously described (P<0.05) 41. The clinical specimens were AR-independent origin: negative AR staining was confirmed by immunohistochemistry by the pathology department.

In the present study, expression vectors of FBI-1 (including FBI-1 or FLAG-FBI-1), FLAG-SP1, siRNA of specificity protein 1 (SP1) (siSP1), and siRNA of FBI-1 (siFBI-1) were used as previously described 16. The full-length sequences of has-pre-miRNA was obtained through chemical synthesis, and the sequences were cloned into lentivirus vectors and prepared as lentivirus particles. For the luciferase reporters, the pentameric sequences of the two SP1-binding sites in the selected region of the miRNA-324-3p promoter region were chemically synthesized and cloned into the pGL4.26 vector (named Luc-1 and Luc-2). The two SP1-binding sites in the selected region of the miRNA-324-3p promoter region (1,000 bp upstream from the hsa-pre-miRNA-324-3p transcription start site) were also amplified by polymerase chain reaction (PCR) for approximately 100 bp upstream and downstream of each binding site. The amplified sequences were cloned into the pGL4.26 vector (termed Luc-3 and Luc-4). Finally, the two 100-bp sequences in the selected region of the miRNA-324-3p promoter that did not contain the SP1-binding sites were cloned into the pGL4.26 vector (termed Luc-5 and Luc-6) as negative control.

### 4.2. Luciferase experiments and quantitative polymerase chain reaction (qPCR)

The effect of FBI-1 on the activation of transcription factor SP1 was determined by luciferase assays (i.e., activation of luciferase reporters) 42,43. PC-3 cells, which were transfected with luciferase reporters (Luc-1-Luc-6) or plasmids (FBI-1, siFBI-1, or siSP1), were harvested for luciferase assays using the kits (Promega Corporation, Madison, WI, USA) according to the instructions provided by the manufacturer and methods described in previous publications 41,44,45. The activation of luciferase reporters was measured by fold-change versus control. For qPCR assays, total RNA samples were extracted from PC-3 cells or LNCaP cells, clinical specimens, or subcutaneous tumors. One-step reverse transcription-qPCR (real-time PCR) was performed in accordance with methods described by the manufacturer and previous publications 46,47. The primers used in the qPCR experiments were: FBI-1, forward sequence: 5'-GCAACATCTGCAAGGTCCGCTT-3', reverse sequence: 5'-TCTTCAGGTCGTAGTTGTGGGC-3'; GX4, forward sequence: 5'-AGTGGATGAAGATCCAACCCAAGG-3', reverse sequence: 5'-GGGCCACACACTTGTGGAGCTAGA-3'; loading control β-actin, forward sequence: 5'-CACCATTGGCAATGAGCGGTTC-3', reverse sequence: 5-AGGTCTTTGCGGAT GTCCACGT-3'. The primers used to amplify the sequences of luciferase reporters were: Luc-3, forward sequence, 5'-ACAAGATGGCGAGG-3', reverse sequence: 5'-AAGTCAAGTGGGGG-3'; Luc4, forward sequence: 5'-CCCCCTCACAGGAAA-3', reverse sequence: 5'-CCCCACTATTTACCCC-3'; Luc5, forward sequence: 5'-TGGGGATGGACGTTT-3'; reverse sequence: 5'-TGCCTCCCAGTTCTT-3'; Luc6, forward sequence: 5'-GTAAGGTCAGATGTGGT-3', reverse sequence: 5'-TGGTGGGAAGGTCAG-3'. The primers of AR (Androgen receptor), LPCAT3 (Lysophosphatidylcholine Acyltransferase 3), TfR1 (transferrin receptor 1), SLC7A11 (Solute Carrier Family 7 Member 11) and FTH1 (Ferritin heavy polypeptide 1) were synthesized and used based on universal primer sequences for each gene provided by OriGene-China Corporation, (Wuxi City, Jiangsu Province, China).

### 4.3. Colony formation and cell migration assays

For colony formation assays, PC-3 cells were stably transfected with vectors, seeded in six-well dishes (in triplicate) (density: 2 × 10^3^ cells per well), and allowed to adhere overnight. During cell culture, the medium was replaced every 3-5 days. Subsequently, colonies were fixed with 4% paraformaldehyde for 25 min and stained with 0.2% crystal violet solution for 30 min at room temperature (25-30°C). Next, the cells were washed, dried, and scanned with an HP Scanjet, USA. For the cell migration assays (wound-healing assays) 46, confluent monolayers of cells were mechanically scratched with a 200-µL pipette tip. Cells were washed thrice with phosphate-buffered saline and treated with dimethyl sulfoxide or compounds in Dulbecco's modified Eagle medium (high glucose) without fetal bovine serum in the presence of mitomycin C (1 µM). Cells were photographed at 0 h and 12 h, and cells that had migrated were counted 46.

### 4.4. Flow cytometry and chemical analysis

The ferroptosis of cultured cells was measured according to the levels of reactive oxygen species (ROS). In tumor tissues, ferroptosis was measured based on the levels of malondialdehyde (MDA) and 4-hydroxynonenal (4-HNE) 47-49. For the measurement of ROS levels, PC-3 cells were transfected with vectors (control, siSP1, FBI-1, or siFBI-1), seeded in six-well dishes (1 × 10^6^ cells per well), and allowed to adhere overnight. Subsequently, the cell culture medium was replaced by diluted 25 µM carboxy-H2DCFDA (88-5930-74; Invitrogen, USA) (2 mL per well), followed by incubation at 37°C for 30 min. Next, the cells were washed thrice with phosphate-buffered saline, and tert-butyl hydroperoxide was added. The ROS levels in PC-3 cells were determined using flow cytometry at a 488-nm excitation wavelength and a 525-nm emission wavelength (FACS Canto II; BD Biosciences, USA). The results are shown as images or quantitative results. For the measurement of ferroptosis in tumor tissues, the levels of MDA and 4-HNE were determined. The relative concentration of MDA in cell and tumor lysates was assessed with a Lipid Peroxidation (MDA) Assay Kit (ab118970; Abcam, UK) according to the instructions provided by the manufacturer. This assay measures the reaction of MDA with thiobarbituric acid (TBA), which generates an MDA-TBA adduct that can be quantified colorimetrically (optical density: 532 nm). A Lipid Peroxidation (4-HNE) Assay Kit (ab238538; Abcam) was used to evaluate the concentration of 4-HNE according to the protocol provided by the manufacturer 47-49.

### 4.5. Western blot

PC-3 cells were obtained in culture. After the transfection was completed in the cells, the cells were collected and lysed using RIPA (Radio Immunoprecipitation Assay), after which 2 x SDS PAGE (polyacrylamide gel electrophoresis) loading buffer was added to the cell samples, mixed thoroughly and then boiled in a water bath for 15 min. After the boiling bath was completed centrifugation was carried out at 4°C 12000 rpm and the supernatant was collected as protein samples. Afterwards, SDS-PAGE gels were prepared and the protein samples were subjected to electrophoresis (80V for 30 min for concentrated gels and 120V for separated gels). After completion of electrophoresis, the samples were transprinted (semi-dry transfer, 15V for 2h) and blocked (blocked with 5% BSA [Bovine serum albumin] in TBST [Tris-Buffered Saline and Tween 20]), and finally the primary and secondary antibodies (SP1 [Cat., No.: sc-420]; FBI-1 [Cat., No.: sc-33683]; β-Actin [Cat., No.: sc-84322]; GAPDH [Cat., No.: sc-47724]; IgG [Cat., No.: sc-2357]) were incubated in sequence and developed by chemiluminescence.

### 4.6. Protein immunoprecipitation and chromatin immunoprecipitation

For protein immunoprecipitation experiments, PC-3 cells were obtained in culture and transfected with FLAG, FLAG-FBI-1 in the cells, after which the cell samples were collected for fragmentation 49,50. The supernatant fraction of the cell suspension was collected for immunoprecipitation experiments with FLAG-beads to isolate the FLAG-FBI-1/SP1 complex from PC-3 cells. The FLAG-beads bound to the FLAG-FBI-1/SP1 complex were subjected to WB assays with antibodies to FLAG-FBI-1 in the complex; antibodies to SP1 were used to detect SP1 in the complex. FLAG-FBI-1 or SP1 in PC-3 cells were also detected and shown to be Input FLAG, Input SP1. For the re-IP experiments, PC-3 cells were obtained in culture, transfected with FLAG, FLAG-SP1 in the cells and then subjected to immunoprecipitation experiments.

For chromatin immunoprecipitation assays, PC-3 cells were cultured and transfected with FBI-1 and siFBI-1 (siFBI1-1 and siFBI1-2), respectively, after which cell samples were collected and fixed and cross-linked with 4% paraformaldehyde and the cells were broken 49,50. The complexes of SP1 and DNA were separated by immunoprecipitation with antibodies to SP1, protein A, and the target sequences of SP1-bound DNA were amplified by PCR. The primers used in the ChIP assays: the forward primer of the input Genomic DNA sequence (the non-specific sequences as control), 5'-AACCTATTAACTCACCCTTGT-3'; the reverse primer of the input Genomic DNA sequence, 5'-CCTCCATTCAAAAGATCTTATTATTTAGCATCTCCT-3'; forward primer to amplify miRNA3a4-3p's promoter, 5'-ATTCGCTTCCTGTTTG-3'; forward primer to amplify miRNA3a4-3p's promoter, 5'-GGAGTGGGAGAAGGA-3'.

### 4.7. *In vivo* tumor model

PC-3 cells were cultured and subcutaneously injected into the medial groin of nude mice aged 5-6 weeks 50-51. The nude mice were housed under specific pathogen-free conditions (all food and bedding were pre-sterilized by ^60^Co-γ irradiation and the pure water used for drinking was autoclaved). After 8-10 weeks of growth, the mice were sacrificed, and subcutaneous tumor tissues were collected. These tumor tissues were analyzed as follows: (1) photographed; (2) the long and short axes of the tumor tissues were accurately measured using vernier calipers to determine the tumor volume (formula: tumor short axis × tumor short axis × tumor long axis); (3) tumor tissues were accurately weighed using a 1/100000 balance; (4) the expression levels of relevant factors were measured within the tumor tissues by qPCR; and (5) the levels of iron death-related factors within the tumor tissues were detected by immunohistochemistry.

### 4.8. Ethics statement

The collection and use of clinical specimens was approved by the Medical Ethics Committee of Qujing Affiliated Hospital of Kunming Medical University. The protocols for the use of human-related materials (i.e., clinical specimens and cell lines) were approved by the ethics review-supervision organization. All experiments were performed according to the Helsinki Declaration (World Health Organization) guidelines. All patients provided written informed consent for their participation in this study.

The protocols for animal experiments were approved by the Animal Ethics Committee of Qujing Affiliated Hospital of Kunming Medical University. All animal experiments in the present study were performed in accordance with the UK Animals (Scientific Procedures) Act of 1986 and associated guidelines.

### 4.9. Statistical analysis

All *in vitro* experiments were performed in triplicate. Statistical differences between variables or groups were assessed using the chi-squared test, two-tailed Student's *t*-test, or one-way analysis of variance. All statistical analyses were performed with the SPSS version 13.0 (SPSS, Chicago, IL, USA) or Prism 6 (GraphPad Inc., San Diego, CA, USA) software. Data are shown as the means ± standard deviations. In all cases, P-values <0.05 denoted statistically significant differences between groups. For the correlation analysis, the RNA levels of GPX4, FBI-1 and miRNA-324-3p in the 32 tumor tissue samples were examined by qPCR. According to the results, scatter plots were drawn using the GPX4 expression levels as the abscissa and the miRNA-324-3p or FBI-1 expression levels as the ordinate; similarly, miRNA-324-3p expression was used as the abscissa and FBI-1 expression was used as the ordinate. Each tumor tissue corresponded to a data point in the scatter plots. Next, linear regression was performed on the data point group, and the regression equation of the correlation between GPX4 with FBI-1, GPX4 with miRNA324-3p, and miRNA-324-3p with FBI-1 was obtained. Correlations were determined based on the slopes of these equations; positive and negative slopes indicated positive and negative correlations, respectively 52.

## Supplementary Material

Supplementary figures.

## Figures and Tables

**Table 1:** The baseline information of patients involved in the presence work

Characters		Number (percentage)
Age (yr), median (range)		63 (37-82)
Histology	Adenocarcinoma GS < 7	4/44 (9.1%)
	Adenocarcinoma GS ≥ 7	37/44 (84.1%)
	Small cell carcinoma	3/44 (6.8%)
Therapeutic strategies to primary tumor	Radiation	10/44 (22.7%)
	Surgery	35/44 (79.5%)
ECOG PS	0	7/44 (15.9)
	1	32/44 (72.7%)
	2	5/44 (11.4%)
