# Supplementary material for: Pro-oncogene FBI-1 inhibits the ferroptosis of prostate carcinoma PC-3 cells via the microRNA-324-3p/GPX4 axis
Source: J Cancer. 2024 Jun 1;15(13):4097–112. doi: 10.7150/jca.96306 (PMC11212100; doi:10.7150/jca.96306)
Supplement: Supplementary file 1 — Supplementary figures. [file jcav15p4097s1.pdf]

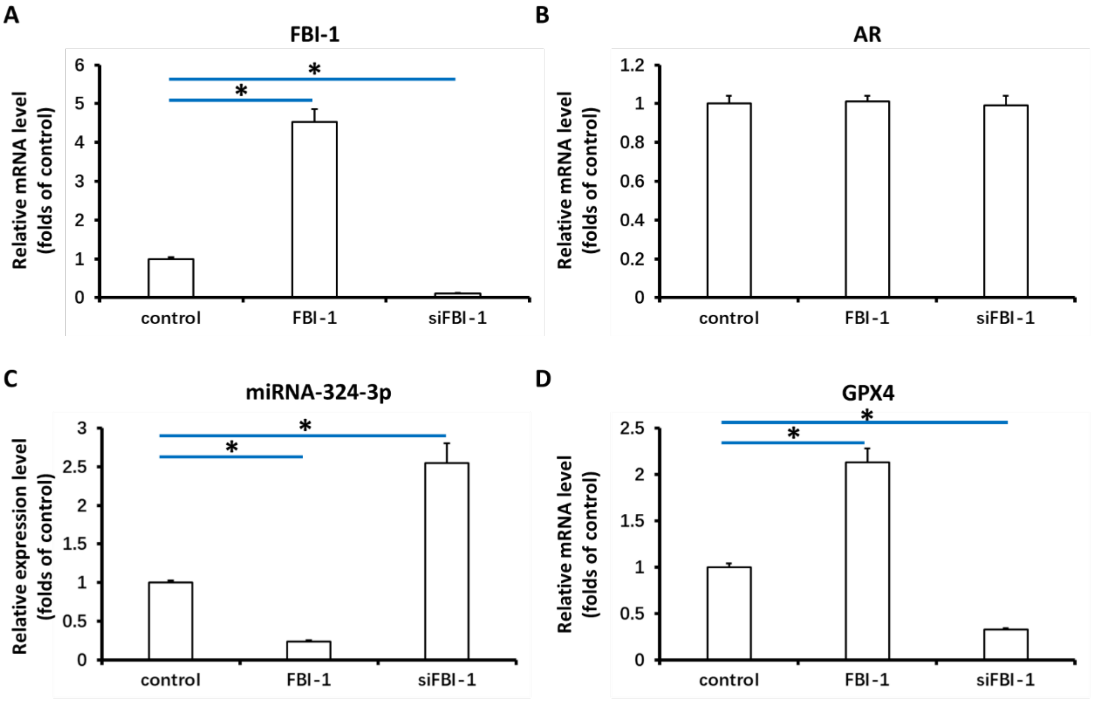

Supplemental Figure 1 The effect of FBI-1 on miRNA-342-3p/GPX4 in LNCaP cells

The LNCaP cells were cultured and transfected with vectors (Control, FBI-1 or siFBI-1 [siFBI1-1]). The expression of FBI-1 (A), AR (B) miRNA-324-3p (C), or GPX4 (D) was examined by qPCR. *P<0.05

Abbreviations: FBI-1, factor that binds to inducer of short transcripts ‐ 1; GPX4, glutathione peroxidase 4; AR: androgen receptor

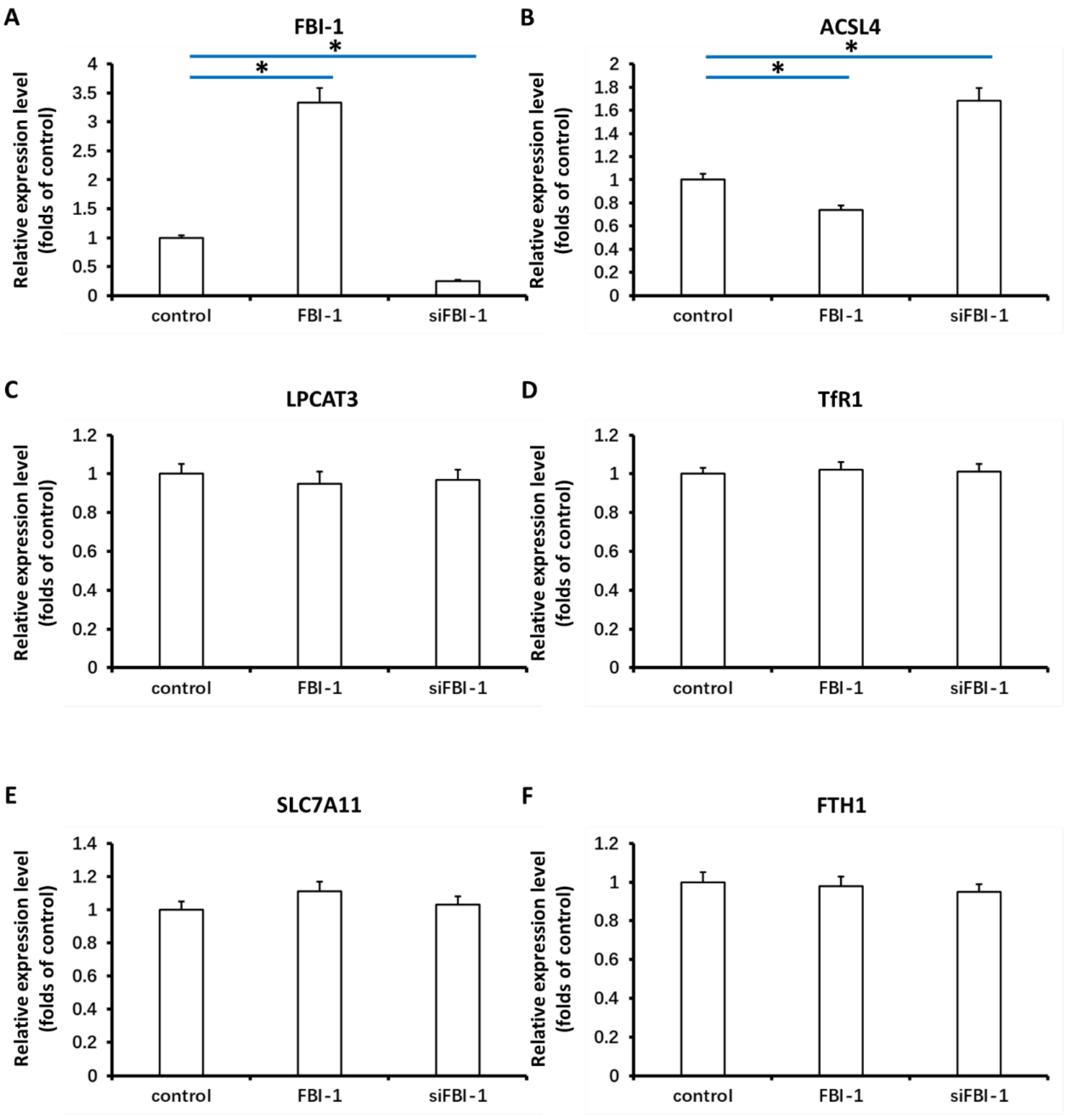

Supplemental Figure 2 The effect of FBI-1 on factors

The LNCaP cells were cultured and transfected with vectors (Control, FBI-1 or siFBI-1 [siFBI1-1]). The expression of FBI-1 (A), ACSL4 (B), LPCAT3 (C), TfR1 (D), SLC7A11 (E), or FTH1 (F) was examined by qPCR. *P<0.05

Abbreviations: FBI-1, factor that binds to inducer of short transcripts - 1; LPCAT3 (Lysophosphatidylcholine Acyltransferase 3); TfR1 (transferrin receptor 1); SLC7A11 (Solute Carrier Family 7 Member 11); FTH1 (Ferritin heavy polypeptide 1)
